# Eclectic Department

**Published:** 1856-09

**Authors:** 


The Cæsarean section was recently successfully performed in Richmond, Va., by Dr. Charles S. Mills, on a negro dwarf, 3 feet 9 inches in height—both mother and child doing well. The operation, it is said, has never before been s..ccessfully performed in that state.—*Ib.*

